# Arch‐supports and plantar fasciitis: A prospective study incorporating patient‐reported outcomes and finite element analysis

**DOI:** 10.1002/jeo2.70732

**Published:** 2026-05-11

**Authors:** Atta Taseh, Omer Subasi, Bedri Karaismailoglu, Kelsey Detels, Samir Ghandour, Carl Rai, Gregory Waryasz, Soheil Ashkani‐Esfahani, Adam Scott Landsman

**Affiliations:** ^1^ Foot & Ankle Research and Innovation Lab (FARIL), Department of Orthopaedic Surgery Mass General Brigham, Harvard Medical School Boston Massachusetts USA; ^2^ Foot & Ankle Division, Department of Orthopaedic Surgery Mass General Brigham, Harvard Medical School Boston Massachusetts USA; ^3^ OMER (Orthopedic and Mechanical Engineering Research) Laboratory Collaborative Network New Haven CT USA; ^4^ Department of Orthopaedic Surgery Mass General Brigham, Harvard Medical School Boston Massachusetts USA

**Keywords:** finite element analysis, foot orthosis, plantar fasciopathy, weight‐bearing CT, weight‐bearing radiograph

## Abstract

**Purpose:**

Arch‐support insoles are used to reduce symptoms in patients with plantar fasciitis. However, the evidence supporting their clinical effectiveness is mixed. This study evaluated the clinical effects of arch‐support insoles by assessing changes in symptom relief (primary outcome), radiographic foot alignment and plantar pressure distribution.

**Methods:**

A single‐arm, prospective study was conducted, and patients aged 18–65 years with a diagnosis of plantar fasciitis were included. Participants received arch‐support insoles at baseline and were evaluated for changes in pain, foot alignment, and plantar pressure distribution. Pain outcomes were measured using Patient‐Reported Outcome Measurement Information System scores for pain intensity and interference at baseline, 4, 8 and 12 weeks. Weight‐bearing computed tomography and x‐rays were obtained at baseline to assess foot alignment with and without insoles. Finite Element analysis was performed using baseline scans to evaluate heel plantar pressure. Statistical tests, Wilcoxon signed‐rank, with statistical significance set at *p* = 0.05. Results are reported as medians with interquartile ranges.

**Results:**

Twenty‐nine patients (median age = 36 years, body mass index = 26 kg/m^2^) were enroled, with a female‐to‐male ratio of 18:11. Pain scores showed improvement from 4 weeks onward (*p* < 0.001). Radiologic assessment revealed increased navicular height on computed tomography (*p* = 0.003) and heightened medial longitudinal arch on radiographs. Finite element analysis (*n* = 12) showed reduced heel stress by 34.7% (peak) and 14.6% (average) (*p* = 0.002). Follow‐up response rates were 87% at Weeks 4 and 12 and 63% at the eighth week.

**Conclusion:**

Our findings underscore the short‐term effect of arch‐support insoles in the treatment of plantar fasciitis. The therapeutic benefit might be correlated with anatomical foot realignment and pressure redistribution, as supported by radiological and biomechanical data. Studies with longer follow‐up and controlled designs are needed to determine the durability and causal nature of these effects beyond 3 months.

**Level of Evidence:**

Level IV.

**Abbreviations**
BMI: body mass index
FAO: foot and ankle offset
FE: finite element
ICC: intraclass correlation coefficient
IMA: intermetatarsal angles
IQR: interquartile range
IRB: institutional review board
M1: first metatarsal bone
M2: second metatarsal bone
PF: plantar fasciitis
PROMIS: Patient‐Reported Outcome Measurement Information System
STL: stereolithography
VAS: visual analogue scale
WB: weight‐bearing
WBCT: weight‐bearing computed tomography

## INTRODUCTION

Plantar fasciitis (PF) is a debilitating musculoskeletal condition causing heel pain, accounting for 1 million visits per year [30]. It could be characterized by both inflammation and degeneration of the plantar fascia due to excessive strain or repetitive stress. Left untreated, PF can result in chronic pain and a diminished quality of life [21]. Common non‐invasive treatments include home‐based stretching and strengthening programs, activity modification, taping, night splints, non‐steroidal anti‐inflammatory drugs and physiotherapy. Arch‐support insoles, including prefabricated and custom insoles, are also frequently prescribed as a non‐invasive intervention. These insoles are designed to reduce strain on the plantar fascia by redistributing plantar pressure and providing structural support to the medial arch of the foot [16]. However, despite their popularity, the effectiveness of arch‐support remains controversial. Some investigations report reductions in pain and improvements in functionality, often measured through validated patient‐reported outcomes such as the Foot Function Index [38]. Other studies indicate minimal, or no added benefit of arch‐supports compared to placebo or alternative treatments [29]. This variability may arise from differences in study design, patient populations, the device used, and the use of subjective and objective measures.

It has been shown that arch‐supports can alter the hindfoot and midfoot alignment in specific pathologies like pes planovalgus, as depicted by radiographic measures [37]. Since anatomical malalignment is also reported in PF, it is unclear whether the efficacy of arch‐supports lies in realigning the foot structure [26]. Furthermore, plantar pressure distribution seems to be altered in PF patients [18]. While some studies have identified a link between radiographic alignment, such as the talus‐first metatarsal (Meary's) angle and plantar pressure distribution, others have failed to observe meaningful associations [20, 36]. However, the relationship between arch‐support‐induced alignment changes and their downstream effect on plantar pressure redistribution in PF remains poorly characterized, in part because conventional radiographs provide only two‐dimensional projections that cannot fully capture the three‐dimensional nature of these structural changes [32]. Weight‐bearing computed tomography (WBCT) enables a three‐dimensional assessment of foot morphology under physiologic loading, while advancements in computational modelling, such as subject‐specific finite element (FE) analysis, allow simulation of the mechanical consequences of observed alignment changes on plantar stress distribution, something not achievable through clinical or radiographic assessment alone [3, 4, 13, 22, 27]. Hence, combining FE analysis with radiological assessments provides a better view of how orthotics work and their role in treating PF.

This study aimed to provide possible mechanistic insight into any observed clinical effects of arch‐supports in the treatment of PF by evaluating (1) symptom relief as measured by the Patient Reported Outcomes Information System (PROMIS) pain interference (primary outcome), (2) radiographic foot alignment and (3) plantar pressure distribution. This multifaceted approach will advance understanding of observed clinical results and generate hypotheses regarding the biomechanical basis of orthotic interventions for future controlled investigations.

## MATERIALS AND METHODS

### Study design and setting

This study was conducted at an academic hospital from June 2022 to March 2024. It was part of a broader single‐arm clinical trial investigating the efficacy of arch‐support insoles on common foot and ankle pathologies, including PF and metatarsalgia. Given the distinct pathophysiology and treatment response profiles of the two conditions, and the observed large effect size (Cohen's *d* = 1.03, 95% confidence interval: 0.57–1.4) in the primary outcome PROMIS Pain Interference for PF patients, this publication reports findings related to PF, while analysis for metatarsalgia is reported separately [34].

### Study participants

Individuals aged 18–65 years diagnosed with PF who could walk up to 0.5 miles within a 24‐h period without difficulty were eligible to be included in the study. Exclusion criteria comprised individuals with a shoe size discrepancy greater than two sizes, a body mass index (BMI) exceeding 35 kg/m^2^, a history of partial or total foot amputation, foot deformities that could interfere with insole fitting, or active ulcers. Additionally, individuals using custom shoes or insoles, assistive walking devices or ankle/foot braces were not eligible. Pregnant participants and those who had undergone a computed tomography scan within 30 days prior to enrolment were also excluded.

### Recruitment and intervention procedures

**Figure 1:**
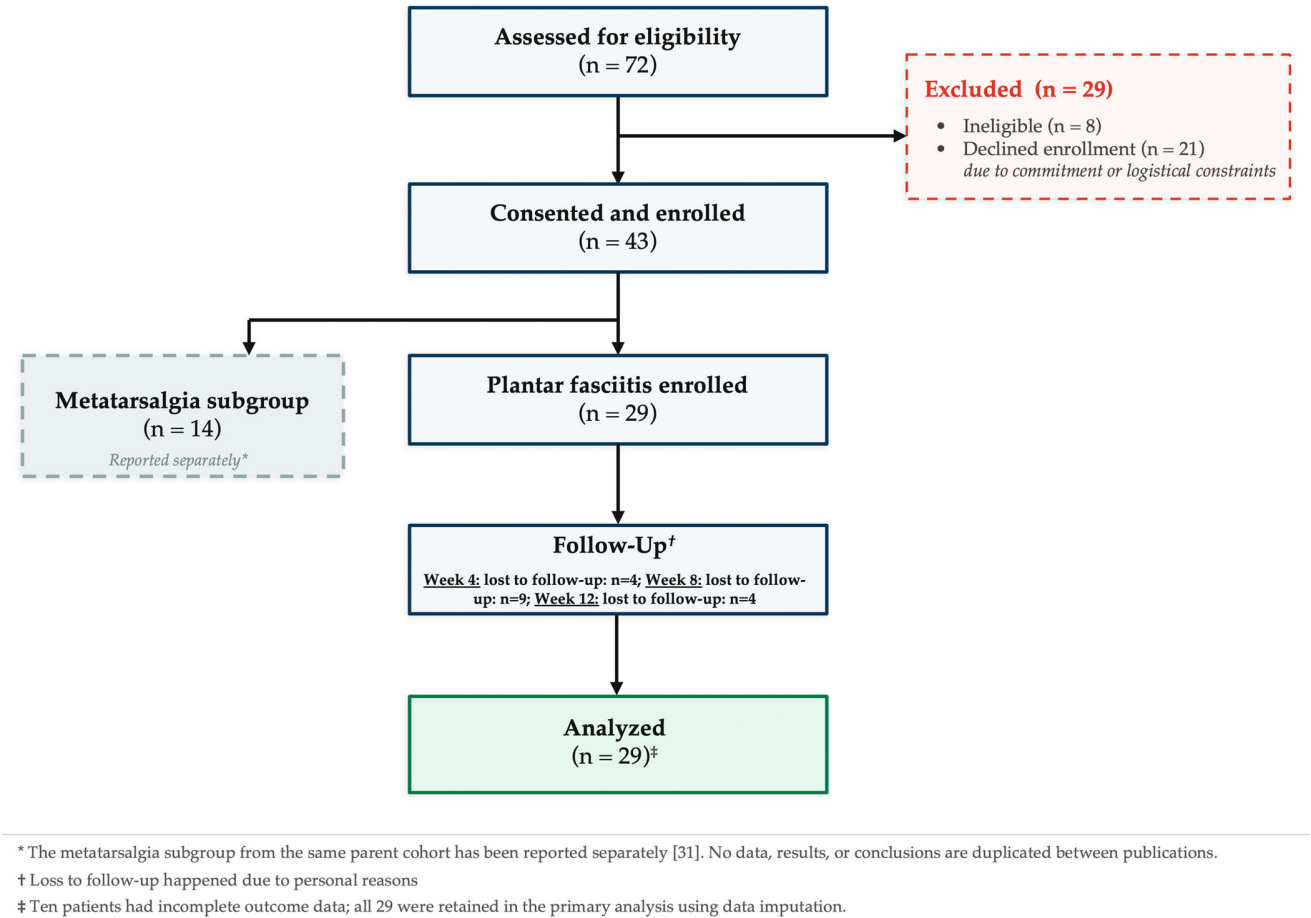
Study recruitment flowchart.

The study was publicly advertised, and interested individuals contacted the research team for further details. The study recruitment flow is shown in Figure 1. Of the 29 included patients, 12 did not attend all study visits due to personal reasons (missing data rates are reported in the Results section).

A senior surgeon (A.L.) assessed patient eligibility. PF was diagnosed based on clinical presentation, including sharp heel pain that was typically worse with the first steps in the morning or after prolonged rest, and exacerbated by standing or activity. Diagnosis was confirmed by localized tenderness at the plantar medial calcaneal tubercle and pain reproduced with passive dorsiflexion of the foot and toes. The ability to walk up to 0.5 miles within a 24‐h period was determined primarily by patient self‐report during the screening interview, and general gait and ambulation were also assessed during the clinical examination to identify potential functional limitations. Furthermore, clinical assessments evaluated possible differential diagnoses, including nerve entrapments, neuropathies, calcaneal injuries, soft tissue injuries, and systemic arthritides. The participants were fitted with appropriately sized partial‐length arch‐support insoles (Good Feet™, Dr.'s Own, LLC.). A graded arch‐support system was employed to provide varying levels of support throughout daily activities, aiming to optimize foot alignment while minimizing discomfort. This system consisted of three insoles made from proprietary blend of medical‐grade polymers such as polypropylene and polyester: (1) the Strengthener, characterized by the highest arch support (arch height to length ratio of 0.17), designed to provide maximum alignment correction; (2) the Maintainer, featuring moderate arch support (arch height to length ratio of 0.15), intended to sustain corrected alignment while allowing more flexibility during routine daily tasks and physical activities such as walking, running, or sports; and (3) the Relaxer, which provided a milder arch‐support (arch height to length ratio of 0.14), specifically for periods of rest and recovery (see Supplementary material, Figure S1A–C). To minimize the effect of different footwear on the study results, all participants were provided with the same shoe and were encouraged to use this as their primary shoe as often as possible (Brooks Ghost, © 2022 Brooks Sports, Inc.). No formal restrictions were placed on additional conservative treatments during the study period to reflect a pragmatic clinical setting; however, participants were instructed to primarily use the study insoles and standardized footwear provided.

Participants were instructed to use the Strengthener initially for at least 30 min daily, gradually increasing usage duration based on individual tolerance and comfort. The Maintainer was recommended for general daily activities to maintain consistent arch alignment throughout dynamic movements. The Relaxer was advised primarily for use during rest periods, particularly in the evenings, to relieve potential arch strain without permitting complete loss of alignment correction. Additionally, participants were guided to use the Relaxer in footwear incompatible with other insoles, such as formal or heeled shoes. This pragmatic approach aimed to promote adherence by balancing therapeutic effectiveness with participants' comfort and practical usability. The patients were also instructed to wear the insoles with the provided shoes whenever possible. Adherence was monitored using a remote weekly self‐reported diary and quantified primarily as the number of days per week each insole type was used.

### Variables and outcome measures

Changes in pain levels were measured using the PROMIS Pain Interference (primary outcome) and Pain Intensity scores at baseline, as well as at follow‐up visits in the 4th, 8th and 12th weeks. To assess the immediate effect of the insoles on foot alignment, WBCT (tube voltage: 120 kV, field of view: 352 × 352 mm^2^, slice thickness: 0.37 mm and dose index: 0.91 mGy) and weight‐bearing (WB) radiographs (anteroposterior and lateral views) were obtained at baseline visit. Imaging was conducted under barefoot conditions, both with and without the Maintainer insoles. The use of the Maintainer insole was only to limit radiation exposure associated with repeated WBCT and radiographic acquisitions. This insole was selected because of its moderate profile, which provides a representative intermediate support profile between the Strengthener and Relaxer insoles. Heel stress distribution changes were measured using WBCT images and FE analysis. Moreover, participants received a weekly diary and were asked to report the type of insole usage each week.

### WBCT and radiographic assessment

To comprehensively evaluate structural changes throughout the foot, including the hindfoot, midfoot, and forefoot, a broad range of linear and angular measurements with validated techniques was utilized, as detailed in Figures S2a–i and S3a–e. WB radiographs were analyzed using eUnity software (version 6.10.2.489) and included measurements such as the intermetatarsal angles (IMAs) between the first and second (M1–M2) metatarsal bones, metatarsal tangent angles, M1 length, hallux valgus angle, talo‐first metatarsal angle and talo‐calcaneal (Kite's) angle on anteroposterior views [5, 8, 31]. Lateral radiographs were used to measure M1 length and declination angle, Meary's angle, and the calcaneal pitch [5, 10, 15, 35]. WBCT images were evaluated using Visage software (Visage Imaging, Version 7.1.18) to derive parameters such as foot and ankle offset (FAO), navicular height, forefoot arch angle, alpha angle and the pronation angle of the first metatarsal [4, 23, 24].

All measurements were performed by experienced orthopaedic researchers—A.T., B.K. and S.G.—with two observers assigned per imaging modality. Each observer independently carried out the measurements twice for every patient: once using images obtained with the insole and once using images obtained without the insole. Blinding of the imaging condition was not feasible because the presence of the insole was visible on both WBCT and radiographic images.

### FE analysis for heel stress distribution

After completion of baseline imaging, using participants' assigned study number, 12 cases were selected using a random selection procedure without consideration of demographic or clinical characteristics in order to minimize selection bias. The patient scans were imported into segmentation software to create the 3D stereolithography (STL) surface models (Materialise Mimics, Materialise Inc.). Given the inter‐comparative nature of the study and the number of simulations to be performed, the bones comprising the foot and ankle were segmented together and exported as a single merged body for each scan. Additionally, the soft tissues encapsulating the skeletal structures were segmented separately. All surface models were cleaned, edited and converted into solid bodies using 3D design software (SpaceClaim).

**Figure 2:**
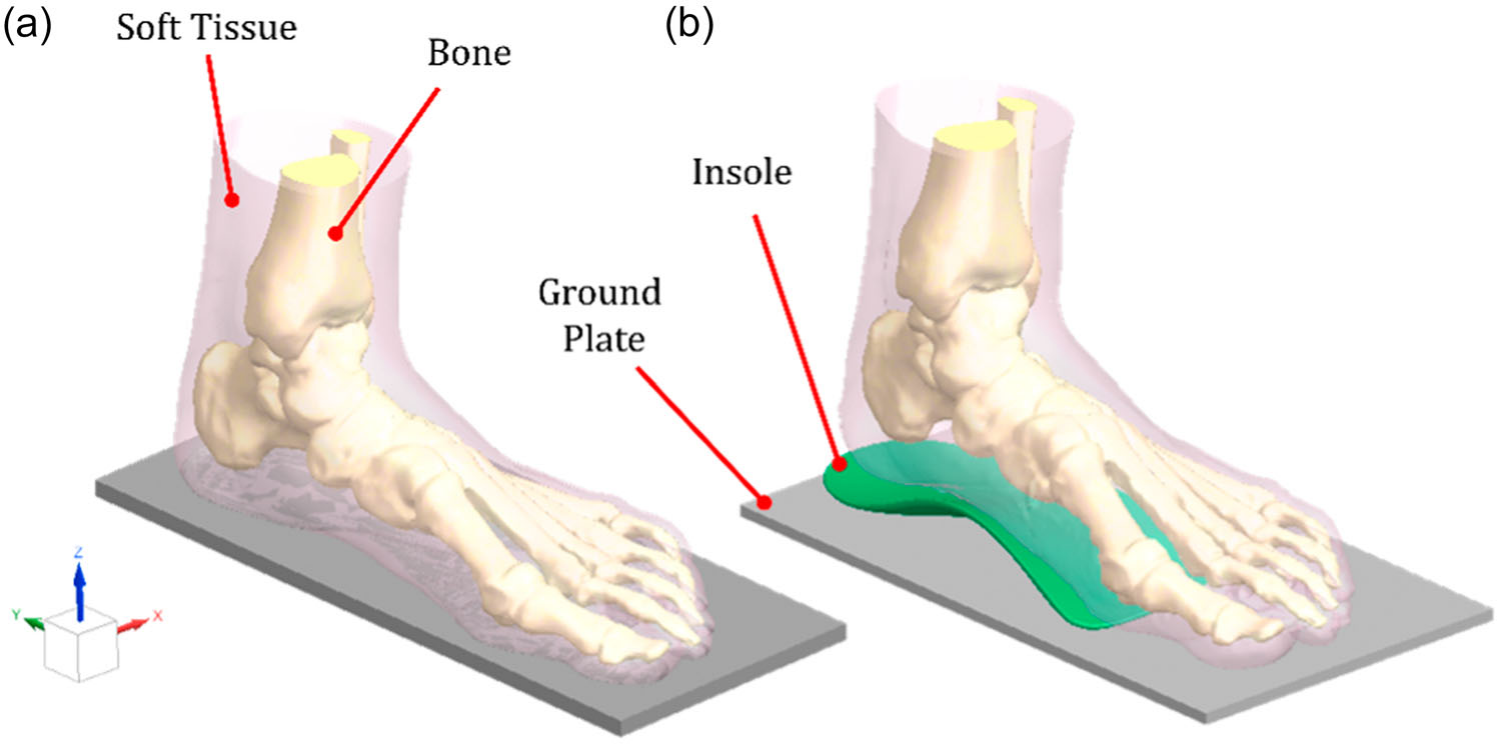
Example of a three‐dimensional computational model of the foot (a) without the insole, (b) with the insole.

The 3D models for simulation were finalized in the design software by bringing together the foot and ankle bones, the soft tissue, the insole and a ground plate. 24 separate models were created for 12 patients without and with insoles, as shown in Figure 2a,b, respectively. For the computational models, the sizes of the insoles were modified for each patient to achieve a perfect fit to the segmentation of the soft tissue.

The biomechanical simulations were performed using the static structural module of a FE analysis software (ANSYS Workbench v21). The material properties used in the simulations are provided in the supplementary material (Table S1) [2, 25, 33]. It was assumed that the bones comprising the foot and ankle consisted solely of cortical bone tissue, and all bodies in the simulation were modelled to behave within the linear elastic range.

**Figure 3:**
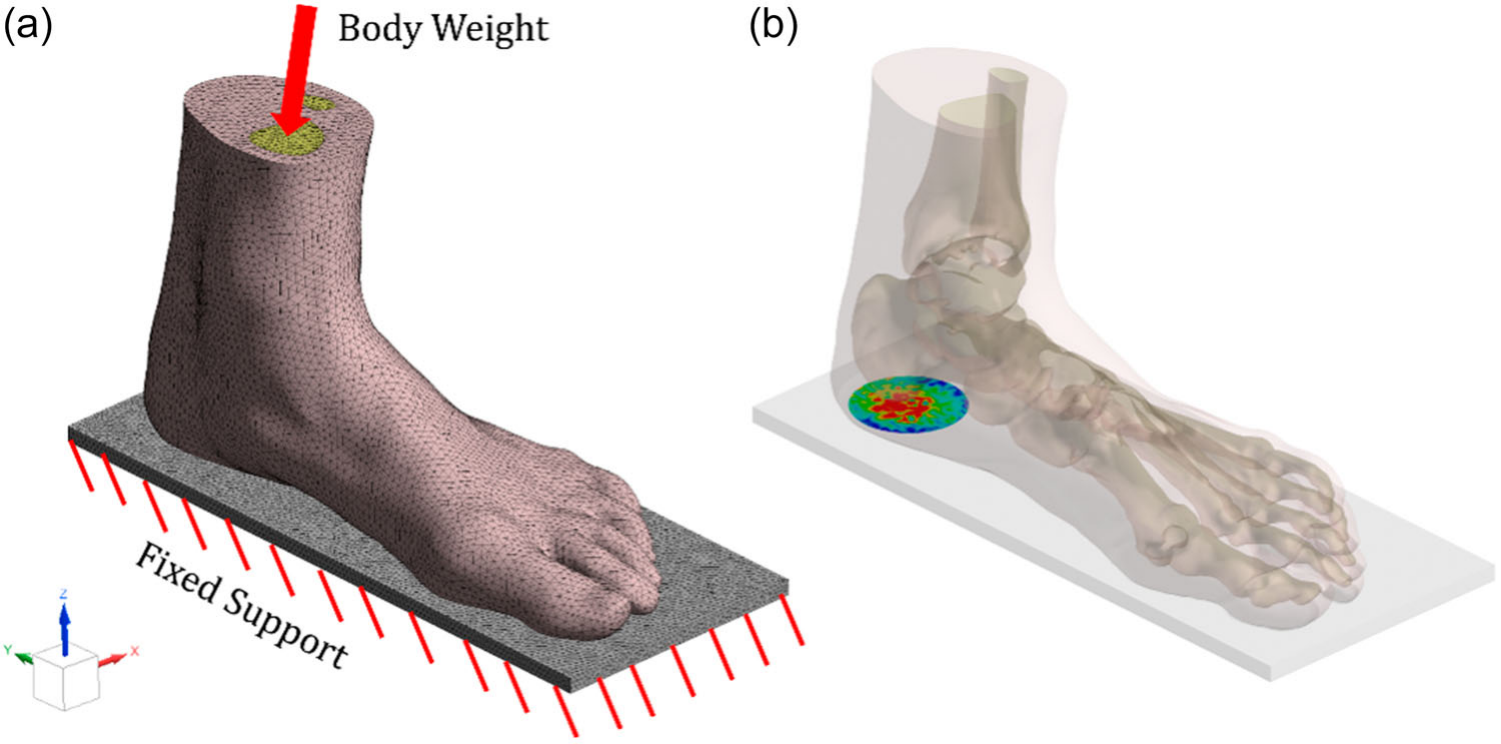
(a) Finite element model showing boundary conditions and mesh structure. (b) Designated heel contact area at the sole‐to‐plate interface for von Mises stress distribution analysis.

The applied boundary conditions for the FE analysis are shown in Figure 3a. The FE parameters used in the simulations are summarized in the supplementary material (Table S2). The foot models were loaded from the top of tibia and fibula with 350 N axial force, which approximately corresponds to the load for two‐legged standing stance for a 70 kg person. The model is fixed from the bottom surface of the ground plate. The contact between the ground plate and the soft tissue is set to frictionless. For the models that include the insole, the insole was bonded to the ground plate from its bottom surface, and the contact between the insole and the soft tissue was once again chosen as frictionless.

The two critical outputs designated for this study were the peak von Mises heel stress ($\sigma_{\mathrm{peak}}$) and average von Mises heel stress ($\sigma_{\mathrm{ave}}$). In order to standardize the heel average stress across different models, a 40‐mm diameter circle with the centre located at the midpoint of the lateral and medial borders of the calcaneus was demarcated as the soft tissue contact area, as shown in Figure 3b. The mesh size was chosen by running a sensitivity analysis, and the tetrahedral mesh size for each body was incrementally decreased to ensure convergence of $\sigma_{\mathrm{peak}}$ and $\sigma_{\mathrm{ave}}$; less than a 5% change for each parameter was chosen as the stop point. The mesh sizes reported in Table S2 were utilized across the whole simulation.

### Statistical analysis

The statistical analysis was performed using SPSS software (IBM SPSS Statistics version 28.0.0.0). Continuous variables were compared across the study time points using the Wilcoxon signed‐rank test, and the values were reported as median and interquartile range (IQR). Cohen's *d* was calculated to measure the effect size of each comparison. Model‐based imputation technique was used to address the missing data. The inter‐observer agreement was measured by means of intraclass correlation coefficient (ICC) based on a two‐way mixed‐effects model, consistency type, with average measures. The ICC values were interpreted as 1, indicating perfect agreement, 0.81 and 0.99 representing excellent agreement, and values from 0.61 to 0.80 signifying substantial agreement [7]. The study power was determined to be 99% with a sample size of 29 patients and a type I error probability of 0.05.

### Ethical considerations

The study protocol was approved by the Institutional Review Board (IRB Massachusetts General Hospital, No. 2022P000291). The risks and benefits of the study procedures were thoroughly explained to all participants, and electronic written informed consent was obtained prior to enrolment.

## RESULTS

A total of 29 patients with a median age of 36 years (IQR: 30.5–49.5) and a median BMI of 26 kg/m^2^ (IQR: 21.5–30) were included in the study. The demographic characteristics revealed a female‐to‐male ratio of 18:11, with the majority of participants being White (*n* = 20), followed by Asian (*n* = 5), individuals with multiple racial backgrounds (*n* = 2), African American (*n* = 1) and those with an unknown racial background (*n* = 1). The response rate to the weekly diary was 86.6%, providing insights into insole usage. Median insole use was 3.5 days/week (IQR: 1.9–4.7) for the Strengthener, 2.9 days/week (IQR: 1.0–3.8) for the Maintainer and 1.5 days/week (IQR: 0.1–3.3) for the Relaxer.

**Figure 4:**
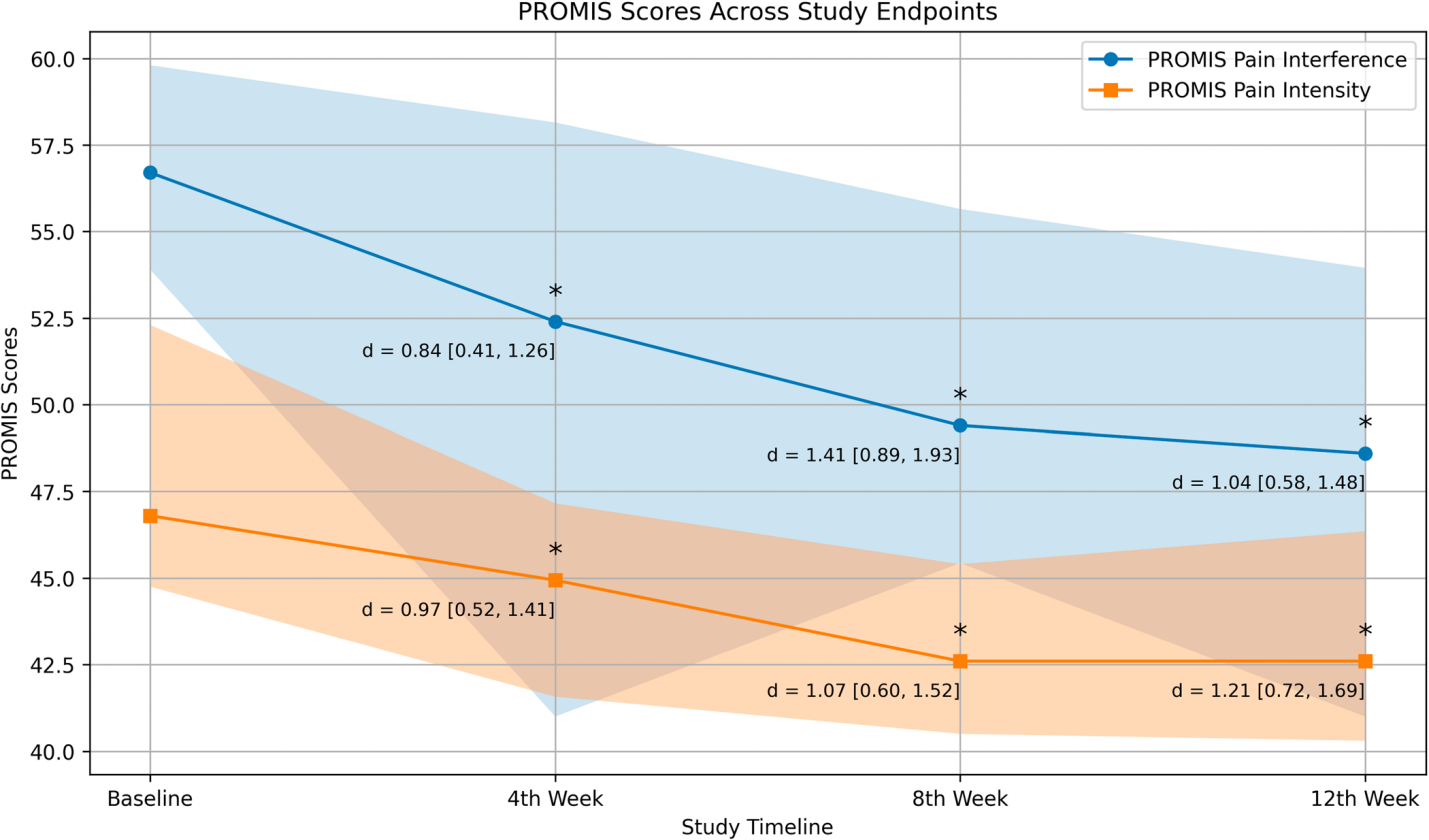
Patient‐Reported Outcomes Measurement Information System (PROMIS) Pain Intensity and Interference scores across the study timeline. Values are presented as medians, with shaded areas representing interquartile ranges. Asterisks (*) indicate significant differences from baseline (p < 0.001), and Cohen's *d* with their associated 95% confidence intervals are presented for each comparison.

PROMIS Pain Intensity and Interference scores improved from the 4‐week follow‐up onwards, showing large effect sizes (*p* < 0.001, Cohen's *d* > 0.8, Figure 4). These improvements were sustained throughout the remainder of the study period. All 29 enroled participants completed the baseline PROMIS questionnaires, yielding a response rate of 100%, whereas follow‐up response rates were 87% at 4th and 12th weeks, and 69% at 8th week. To assess potential attrition bias, baseline characteristics were compared between participants with complete follow‐up data (*n* = 19) and those with one or more missing PROMIS assessments (*n* = 10). Groups did not differ significantly in sex (*p* = 0.58), BMI (median 26.2 [IQR: 22.8–30.0] vs. 22.7 [IQR: 20.8–26.6] kg/m^2^; *p* = 0.18), baseline PROMIS Pain Intensity (median 46.8 [IQR: 43.8–53.7] vs. 48.0 [IQR: 44.2–51.1]; *p* = 0.62), or baseline PROMIS Pain Interference (median 57.3 [IQR: 55.0–60.4] vs. 54.6 [IQR: 51.3–57.2]; *p* = 0.06). Participants with missing data were, however, significantly younger than completers (median 30.5 [IQR: 28.0–37.0] vs. 45.0 [IQR: 34.0–53.0] years; *p* = 0.01).

WBCT assessments demonstrated an increase in navicular height with the use of arch‐support insoles (*p* = 0.003; Table 1). Additional radiographic assessment indicated alterations in foot alignment, as evidenced by increased M1 declination angle (*p* < 0.001), calcaneal pitch (*p* = 0.007), Kite's angle (*p* = 0.03) and Meary's angle (*p* = 0.002; Table 2). The inter‐rater reliability of these measurements is summarized in Tables 1 and 2. The medical images from 28 participants were analyzed as one of the participants could not attend the imaging appointment.

**Table 1:** Assessment of foot alignment using WBCT, performed with (W) and without (WO) insoles.

Measurements	Median (IQR)	*p*	ICC^a^	95% CI
FAO W (%)	3.2 (0.7, 5)	0.86	0.93	0.85–0.97
FAO WO (%)	2.8 (0.91, 5.4)		0.95	0.88–0.97
Navicular height W (mm)	33.4 (30.4, 38)	**0.003**	0.94	0.87–0.97
Navicular height WO (mm)	31.5 (28.4, 37)		0.95	0.88–0.98
Alpha angle W (degrees)	13.8 (6.6, 16.4)	0.19	0.90	0.80–0.95
Alpha angle WO (degrees)	13.6 (5.7, 16.4)		0.92	0.82–0.96
Forefoot arch angle W (degrees)	9.9 (7.3, 14)	0.06	0.96	0.90–0.98
Forefoot arch angle WO (degrees)	10.6 (7.6, 14.4)		0.96	0.91–0.98
M1 pronation angle W (degrees)	7.2 (4.4, 12)	0.13	0.89	0.75–0.94
M1 pronation angle WO (degrees)	7 (2.8, 11)		0.81	0.60–0.91

*Note*: Statistically significant values are presented in bold.

Abbreviations: CI, confidence interval; FAO, foot and ankle offset; ICC, intraclass correlation coefficient; IQR, interquartile range; W, with insole; WBCT, weight‐bearing computed tomography; WO, without insole.

^a^ Two‐way mixed effects model.

**Table 2:** Assessment of foot alignment using weight‐bearing radiographs, performed with and without arch‐support insoles.

Measurements	Median (IQR)	*p*	ICC^a^	95% CI
HVA W (degrees)	10.25 (6.12, 13.75)	0.98	0.93	0.84–0.96
HVA WO (degrees)	9.7 (6.4, 13.5)		0.96	0.92–0.98
M1‐M2 IMA W (degrees)	9.25 (7.12, 11)	0.3	0.89	0.76–0.95
M1‐M2 IMA WO (degrees)	9 (8, 11)		0.78	0.53–0.90
M1 declination W (degrees)	24 (21.6, 25.5)	**<0.001**	0.95	0.90–0.98
M1 declination WO (degrees)	21.3 (19, 24)		0.88	0.75–0.94
M1‐M2 tangent W (degrees)	5 (−0.4, 8.4)	0.4	0.95	0.88–0.97
M1‐M2 tangent WO (degrees)	5.3 (−0.9, 7.5)		0.94	0.88–0.97
M2‐M3 tangent W (degrees)	15.8 (11.4, 18.4)	0.34	0.77	0.51–0.90
M2‐M3 tangent WO (degrees)	14.5 (11.9, 20)		0.82	0.61–0.92
M2‐M4 tangent W (degrees)	23.8 (20.8, 27.9)	0.21	0.83	0.62–0.92
M2‐M4 tangent WO (degrees)	23.5 (21.6, 29)		0.93	0.84–0.96
M2‐M5 tangent W (degrees)	31 (30.3, 35)	0.53	0.90	0.80–0.95
M2‐M5 tangent WO (degrees)	31.8 (29.3, 34.5)		0.92	0.83–0.96
Lat Meary's angle W (degrees)	0.8 (−4.5, 7.5)	**0.002**	0.86	0.70–0.93
Lat Meary's angle WO (degrees)	−1.5 (−8.9, 6.75)		0.88	0.75–0.96
AP Meary's angle W (degrees)	16 (8.5, 22.1)	0.64	0.96	0.91–0.98
AP Meary's angle WO (degrees)	15.5 (8.3, 20.8)		0.90	0.80–0.95
Calcaneal pitch W (degrees)	18 (15.6, 19.5)	**0.007**	0.96	0.85–0.97
Calcaneal pitch WO (degrees)	18 (15, 19.5)		0.91	0.81–0.96
Kite's angle W (degrees)	25.5 (21.1, 30.1)	**0.03**	0.83	0.63–0.92
Kite's angle WO (degrees)	27 (23, 32)		0.88	0.75–0.95
AP M1 length W (mm)	68 (64, 72.5)	0.19	0.99	0.98–0.99
AP M1 length WO (mm)	68.2 (65.4, 72.7)		0.97	0.93–0.99
Lat M1 length W (mm)	71.1 (67.7, 75)	0.7	0.99	0.98–0.99
Lat M1 length WO (mm)	71.3 (66.8, 75.7)		0.99	0.98–0.99

*Note*: Statistically significant values are presented in bold.

Abbreviations: AP, anteroposterior; CI, confidence interval; HVA, hallux valgus angle; ICC, intraclass correlation coefficient; IMA, intermetatarsal angle; IQR, interquartile range; Lat, lateral; M#, metatarsal bone number; W, with insole; WO, without insole.

^a^ Two‐way mixed effects model.

**Figure 5:**
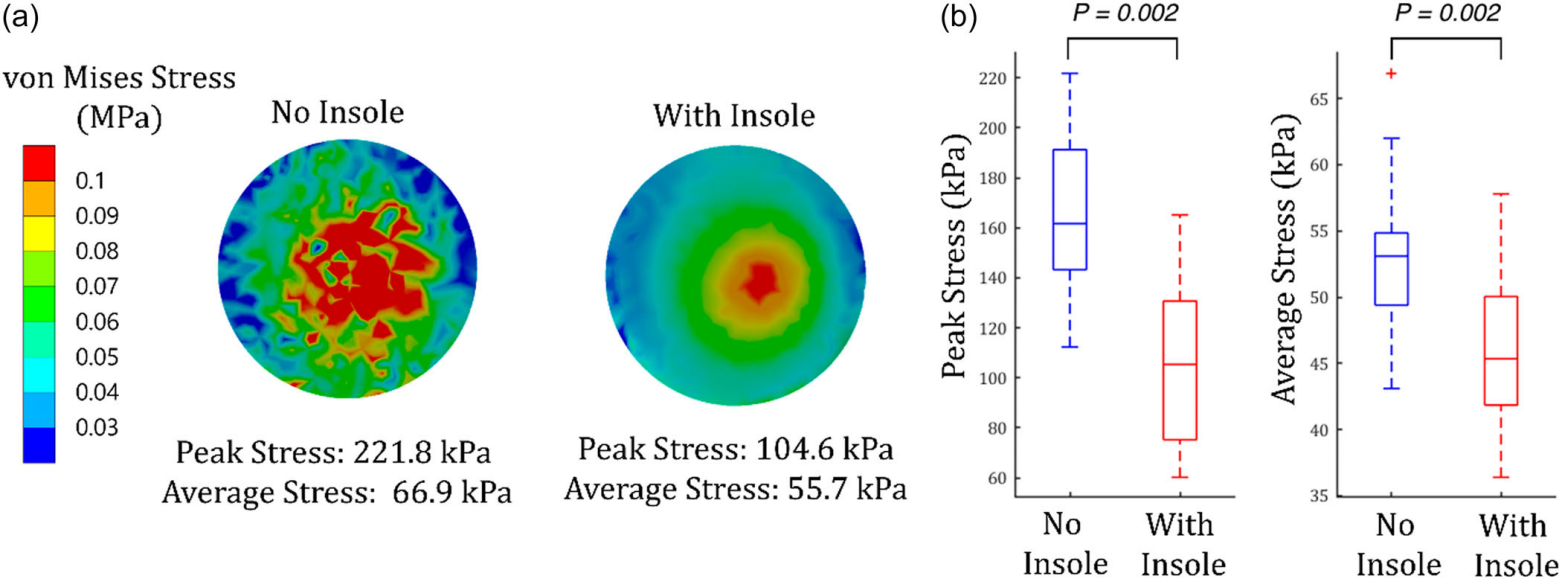
Finite element analysis (FEA) of heel stress distribution in patients with plantar fasciitis with and without arch‐support insoles. (a) Example of the analysis for a single patient and (b) boxplot presentation of the peak and average heel stress distribution for a sample of 12 patients from the study population.

FE analysis was performed on WBCT data from a randomly selected subset of 12 patients with a median age of 36 years (IQR: 29.2–47.5; *p* = 0.64 compared to the main cohort), a BMI of 25.6 kg/m^2^ (IQR: 21.5–26.6; *p* = 0.35), a female‐to‐male ratio of 5:7 (Fisher's exact *p* = 0.12), and consisting of 7 White participants, 4 Asian participants, 1 African American participant and 1 participant of multiple racial backgrounds. The analysis demonstrated reductions in peak and average heel stress distributions by 34.7% and 14.6%, respectively, with the use of arch‐support insoles (*p* = 0.002; Figure 5a,b).

## DISCUSSION

This study adopted a multidisciplinary approach to evaluate the clinical effects of arch‐support insoles in the treatment of PF and to provide a possible biomechanical context for the observed findings. Radiologic assessments revealed immediate structural changes measured by navicular height and other forefoot and hindfoot measures. FE simulations showed a reduction of peak and average stress in the heel area. These findings were accompanied by symptomatic pain relief that persisted over the twelve‐week course of the study.

This study has several methodological strengths, most notably the adoption of a comprehensive approach that integrates clinical outcomes with advanced imaging modalities, such as WBCT and FE analysis. However, as a single‐arm trial without a control group, the study design limits the ability to isolate the intervention's true efficacy from natural disease progression. Attrition analysis revealed that participants with missing data were significantly younger than completers, while baseline pain scores and other demographic characteristics did not differ significantly between groups. This age‐related differential dropout may reflect lifestyle or scheduling factors rather than differential treatment response, though this interpretation should be made cautiously given the modest sample size. Concomitant conservative treatments were not systematically recorded, which may represent a potential source of confounding for the observed clinical outcomes. The use of standardized footwear and a graded arch‐support protocol was an intentional design feature aimed at reducing variability in shoe characteristics and reflecting pragmatic clinical use. Although these choices improve real‐world applicability, they also limit the ability to isolate the effect of a single insole condition. The FE analysis used simplified modelling assumptions, including standardized axial loading, linear elastic soft tissue properties, and frictionless contact interfaces, to enable consistent within‐subject comparison of insole effects. These choices improve model stability and comparability but do not fully reproduce patient‐specific or dynamic loading conditions. In addition, the model was not validated against in vivo plantar pressure data. Therefore, the FE findings should be interpreted as mechanistic support for the observed clinical trends. Future work should expand the cohort size and consider including a control group to strengthen the generalizability of the findings. Additionally, the FE group could be selected based on clinical outcomes to strengthen the association between findings and clinical symptoms.

There is a growing body of literature supporting the use of insoles as a conservative treatment for PF. Compared with other conservative measures such as stretching, physiotherapy, or night splints, their conceptual role is more specifically directed toward mechanical support and alignment modifications. Yildiz et al. reported improvements in pain scores, measured by the visual analogue scale (VAS), following the use of custom‐made insoles designed to provide arch‐support [40]. These outcomes were comparable to those of groups receiving complementary treatments such as exercise and manual therapy. Other studies have investigated the comparative efficacy of custom versus prefabricated insoles. Wrobel et al. observed similar improvements in VAS pain scores between custom and prefabricated insoles, with no difference between groups (*p* = 0.26) [39]. Similarly, Landorf et al. reported comparable improvements in function at three months, as assessed by the Foot Health Status Questionnaire [17]. However, while functional gains were evident in both groups, pain relief was observed only in the prefabricated insole group when compared to a sham intervention (mean difference = 8.7, *p* = 0.05). Our study demonstrated pain relief as indicated by PROMIS Pain Intensity and Interference scores beginning at Week 4 and sustained throughout the study period. The implementation of a prefabricated graded arch‐support system in this study allowed dynamic support tailored to varying activity levels. This adaptive support potentially enhanced comfort, improved compliance, and thereby contributed to better overall patient outcomes.

Foot posture, including midfoot and hindfoot alignment, has been previously associated with PF. Johannsen et al. conducted a case‐control study measuring the Foot Posture Index in 216 patients and controls, finding associations between PF and both hyperpronation (odds ratio: 5.4, *p* < 0.001) and hypopronation (odds ratio: 2.9, *p* = 0.01) [12]. Similarly, Prichasuk and Subhadrabandhu reported lower calcaneal pitch angles on radiographs of patients experiencing heel pain [28]. Our study further supports these findings, demonstrating notable midfoot alterations characterized by increased Meary's and first metatarsal declination angles on radiographs, alongside elevated navicular heights as observed on WBCT. Additionally, hindfoot changes were identified through an increased calcaneal pitch and decreased Kite's angle, indicating possible elevation and varus alignment shifts. Collectively, these radiographic improvements highlight the efficacy of arch‐support insoles in restoring medial longitudinal arch alignment. These findings provide objective evidence that arch‐support insoles can alter foot posture in a manner consistent with their intended biomechanical function. They align with prior biomechanical work by Kogler et al., suggesting that correcting medial longitudinal arch alignment reduces stress on the plantar fascia by lowering tensile loads [14]. Although the study design with baseline imaging does not allow direct confirmation of the full temporal pathway linking alignment change to pain reduction, the radiographic findings add important mechanistic context to the observed clinical benefits. Worth mentioning that imaging was performed under barefoot conditions, trying to improve the interpretability of the insole‐specific effects, but it may limit direct generalizability to routine in‐shoe use.

Various footwear and insole designs have been investigated in the literature to reduce plantar pressures in the heel region among patients with PF [11, 18]. Goske et al. demonstrated that insoles fully conforming to heel geometry can decrease peak plantar pressures by up to 44% compared to barefoot conditions [9]. Similarly, an FE analysis by Chen et al. reported reductions of 36% in peak heel pressures and 56% in average heel pressures when total contact insoles were used [6]. Consistent with these findings, our study observed a decrease in plantar pressures with the use of arch‐support insoles. One plausible explanation for this reduction is the altered hindfoot alignment, as evidenced by the increased calcaneal pitch and decreased Kite's angle. However, this mechanism is challenged by findings from a study by Lee et al., which reported elevated heel pressures in PF patients regardless of whether they had flatfoot or neutral foot alignment, casting doubt on the role of foot posture alone in determining plantar pressures [19]. Another explanation could be the distribution of ground reactive forces over a larger surface area, i.e. the arch is now carrying some of the load that was previously only carried by the heel, lateral border of the midfoot, and forefoot. This concept has been well established in the diabetic foot literature, reduced forefoot pressures with the use of custom insoles [1]. Moreover, increased contact area facilitated by these insoles has been inversely associated with plantar fascia stress [11]. This decrease in plantar fascia stress could theoretically aid in tissue healing, underscoring the potential clinical importance of these biomechanical changes.

In conclusion, this study demonstrates a statistically significant short‐term reduction in pain with the use of arch‐support insoles in patients with PF. Pain relief was observed by 4 weeks and sustained through 12 weeks. These benefits might be related to structural realignment of the foot, as shown by advanced imaging, and reduction in heel stress, as evidenced by FE analysis. Together, these findings highlight the value of combining clinical, radiological and computational approaches to evaluate and optimize conservative interventions for musculoskeletal conditions like PF. Although the findings are encouraging, further controlled studies are required to assess whether the benefits are maintained beyond the 3‐month timeframe.

## AUTHOR CONTRIBUTIONS

Study design, patient recruitment, data collection, analysis and manuscript drafting: Atta Taseh. Finite element analysis, biomechanical modelling, data interpretation and manuscript drafting: Omer Subasi. Radiologic measurements and analysis, manuscript drafting: Bedri Karaismailoglu. Patient recruitment and data collection: Kelsey Detels. Radiologic assessment and analysis, manuscript editing: Samir Ghandour. Finite element analysis and manuscript editing: Carl Rai. Study supervision and manuscript revision: Gregory Waryasz. Study design, statistical analysis and manuscript revision: Soheil Ashkani‐Esfahani. Study conception and supervision, clinical assessment, data collection and manuscript revision: Adam Scott Landsman. All authors approved the final manuscript and agreed to be accountable for all aspects of the work.

## CONFLICT OF INTEREST STATEMENT

The authors declare no conflicts of interest.

## ETHICS STATEMENT

The study protocol was approved by the Mass General Brigham Institutional Review Board (IRB No. 2022P000291). Written informed consent was obtained from all patients prior to study enrolment.

## Supporting information


Supporting File 1

Supporting File 2

Supporting File 3

Supporting File 4

Supporting File 5

Supporting File 6

## Data Availability

De‐identified study data may be made available upon reasonable request, contingent upon approval by the Institutional Review Board.
