# Supplementary material for: Arch‐supports and plantar fasciitis: A prospective study incorporating patient‐reported outcomes and finite element analysis
Source: J Exp Orthop. 2026 May 11;13(2):e70732. doi: 10.1002/jeo2.70732 (PMC13161469; doi:10.1002/jeo2.70732)
Supplement: Supplementary file 1 — Supporting File 1 [file JEO2-13-e70732-s005.doc]

STROBE Statement—checklist of items that should be included in reports of observational studies

|  | Item No | Recommendation |
| --- | --- | --- |
| **Title and abstract** | 1 | (*a*) Indicate the study’s design with a commonly used term in the title or the abstract  Line 7 |
|  |  | (*b*) Provide in the abstract an informative and balanced summary of what was done and what was found Lines 2-26 |
| Introduction | | |
| Background/rationale | 2 | Explain the scientific background and rationale for the investigation being reported Lines 29-54 |
| Objectives | 3 | State specific objectives, including any prespecified hypotheses Lines 55-59 |
| Methods | | |
| Study design | 4 | Present key elements of study design early in the paper Lines 61-78 |
| Setting | 5 | Describe the setting, locations, and relevant dates, including periods of recruitment, exposure, follow-up, and data collection Lines 79-108 |
| Participants | 6 | (*a*) *Cohort study*—Give the eligibility criteria, and the sources and methods of selection of participants. Describe methods of follow-up  *Case-control study*—Give the eligibility criteria, and the sources and methods of case ascertainment and control selection. Give the rationale for the choice of cases and controls  *Cross-sectional study*—Give the eligibility criteria, and the sources and methods of selection of participants  *Single-arm clinical trial___* relevant information about the participants are provided in Lines 70-78 |
|  |  | (*b*) *Cohort study*—For matched studies, give matching criteria and number of exposed and unexposed  *Case-control study*—For matched studies, give matching criteria and the number of controls per case |
| Variables | 7 | Clearly define all outcomes, exposures, predictors, potential confounders, and effect modifiers. Give diagnostic criteria, if applicable Lines 109-118 |
| Data sources/ measurement | 8* | For each variable of interest, give sources of data and details of methods of assessment (measurement). Describe comparability of assessment methods if there is more than one group Lines:119-170 |
| Bias | 9 | Describe any efforts to address potential sources of bias Lines 60-184  **Selection bias:** Clear inclusion and exclusion criteria to reduce confounding (BMI, deformities, custom insoles, recent CT, etc.); Standardized diagnostic criteria for PF, including differential diagnosis screening.  **Measurement bias:** Imaging assessments performed by two independent observers, each repeating measurements twice; Inter-observer reliability quantified with intraclass correlation coefficients (ICC); Use of validated radiographic and WBCT parameters; FE analysis mesh sensitivity testing to ensure numerical convergence.  **Confounding bias:** Standardization of footwear by providing identical shoes to all participants; Imaging performed under controlled barefoot conditions, with and without insoles.  **Reporting / attrition bias:** Weekly adherence diaries to monitor insole usage; Model-based imputation used to address missing data instead of complete-case analysis.  **Statistical bias:** Use of appropriate non-parametric test (Wilcoxon Signed-Rank) for small sample sizes; Reporting of effect sizes (Cohen’s d) alongside p-values; Power analysis demonstrating adequate sample size. |
| Study size | 10 | Explain how the study size was arrived at Lines 179-180 |
| Quantitative variables | 11 | Explain how quantitative variables were handled in the analyses. If applicable, describe which groupings were chosen and why N/A |
| Statistical methods | 12 | (*a*) Describe all statistical methods, including those used to control for confounding Lines 172-180 |
|  |  | (*b*) Describe any methods used to examine subgroups and interactions Lines 172-180 |
|  |  | (*c*) Explain how missing data were addressed Lines172-180 |
|  |  | (*d*) *Cohort study*—If applicable, explain how loss to follow-up was addressed Lines 172-180  *Case-control study*—If applicable, explain how matching of cases and controls was addressed  *Cross-sectional study*—If applicable, describe analytical methods taking account of sampling strategy |
|  |  | (*e*) Describe any sensitivity analyses Lines 167 |

Continued on next page

| Results | | |
| --- | --- | --- |
| Participants | 13* | (a) Report numbers of individuals at each stage of study—eg numbers potentially eligible, examined for eligibility, confirmed eligible, included in the study, completing follow-up, and analysed Lines 186-192, 196-197, 202-203 |
|  |  | (b) Give reasons for non-participation at each stage Lines 202-203 |
|  |  | (c) Consider use of a flow diagram |
| Descriptive data | 14* | (a) Give characteristics of study participants (eg demographic, clinical, social) and information on exposures and potential confounders Lines 186-192 |
|  |  | (b) Indicate number of participants with missing data for each variable of interest Lines 186-192, 196-197, 202-203 |
|  |  | (c) *Cohort study*—Summarise follow-up time (eg, average and total amount) Lines 196-197 |
| Outcome data | 15* | *Cohort study*—Report numbers of outcome events or summary measures over time Lines 192-209 |
|  |  | *Case-control study—*Report numbers in each exposure category, or summary measures of exposure |
|  |  | *Cross-sectional study—*Report numbers of outcome events or summary measures |
| Main results | 16 | (*a*) Give unadjusted estimates and, if applicable, confounder-adjusted estimates and their precision (eg, 95% confidence interval). Make clear which confounders were adjusted for and why they were included |
|  |  | (*b*) Report category boundaries when continuous variables were categorized |
|  |  | (*c*) If relevant, consider translating estimates of relative risk into absolute risk for a meaningful time period N/A |
| Other analyses | 17 | Report other analyses done—eg analyses of subgroups and interactions, and sensitivity analyses N/A |
| Discussion | | |
| Key results | 18 | Summarise key results with reference to study objectives Lines 211-216 |
| Limitations | 19 | Discuss limitations of the study, taking into account sources of potential bias or imprecision. Discuss both direction and magnitude of any potential bias Lines 217-226 |
| Interpretation | 20 | Give a cautious overall interpretation of results considering objectives, limitations, multiplicity of analyses, results from similar studies, and other relevant evidence Lines 227-273 |
| Generalisability | 21 | Discuss the generalisability (external validity) of the study results Lines 227-273 |
| Other information | | |
| Funding | 22 | Give the source of funding and the role of the funders for the present study and, if applicable, for the original study on which the present article is based Title page |

*Give information separately for cases and controls in case-control studies and, if applicable, for exposed and unexposed groups in cohort and cross-sectional studies.

**Note:** An Explanation and Elaboration article discusses each checklist item and gives methodological background and published examples of transparent reporting. The STROBE checklist is best used in conjunction with this article (freely available on the Web sites of PLoS Medicine at http://www.plosmedicine.org/, Annals of Internal Medicine at http://www.annals.org/, and Epidemiology at http://www.epidem.com/). Information on the STROBE Initiative is available at www.strobe-statement.org.
