# Supplementary material for: Arch‐supports and plantar fasciitis: A prospective study incorporating patient‐reported outcomes and finite element analysis
Source: J Exp Orthop. 2026 May 11;13(2):e70732. doi: 10.1002/jeo2.70732 (PMC13161469; doi:10.1002/jeo2.70732)
Supplement: Supplementary file 2 — Supporting File 2 [file JEO2-13-e70732-s001.docx]

| **Table S1.** Material properties utilized in the finite element simulations | | | | |
| --- | --- | --- | --- | --- |
| **Body** | **Material** | **Behavior** | **Elastic Modulus (MPa)** | **Poisson’s Ratio** |
| **Bone** | Cortical Tissue | Isotropic Linear Elasticity | 7300 | 0.3 |
| **Skin** | Soft Tissue |  | 1.15 | 0.49 |
| **Insole** | TPU |  | 11 | 0.45 |
| **Ground Plate** | Stainless Steel |  | 200,000 | 0.27 |
| Abbreviations: TPU, thermoplastic polyurethane | | | | |
