# Supplementary material for: Arch‐supports and plantar fasciitis: A prospective study incorporating patient‐reported outcomes and finite element analysis
Source: J Exp Orthop. 2026 May 11;13(2):e70732. doi: 10.1002/jeo2.70732 (PMC13161469; doi:10.1002/jeo2.70732)
Supplement: Supplementary file 3 — Supporting File 3 [file JEO2-13-e70732-s006.docx]

| **Table S2.** Parameters utilized in the finite element simulations. | |
| --- | --- |
| **Parameter** | **Values** |
| **Mesh Type** | Tetrahedral |
| **Mesh Sizing** | Soft Tissue: 3 mm |
|  | Bone: 2 mm |
|  | Plate/Insole: 2 mm |
| **Min-Max FE # (10^6^)** | 1.00-1.17 |
| **Min-Max FE Node # (10^6^)** | 1.58-1.78 |
| **Bone-Soft Tissue Contact** | Bonded |
| **Soft Tissue- Plate/Insole Contact** | Frictionless |
| Abbreviation: FE, finite element | |
