# Supplementary figures and images for: Arch‐supports and plantar fasciitis: A prospective study incorporating patient‐reported outcomes and finite element analysis
Source: J Exp Orthop. 2026 May 11;13(2):e70732. doi: 10.1002/jeo2.70732 (PMC13161469; doi:10.1002/jeo2.70732)

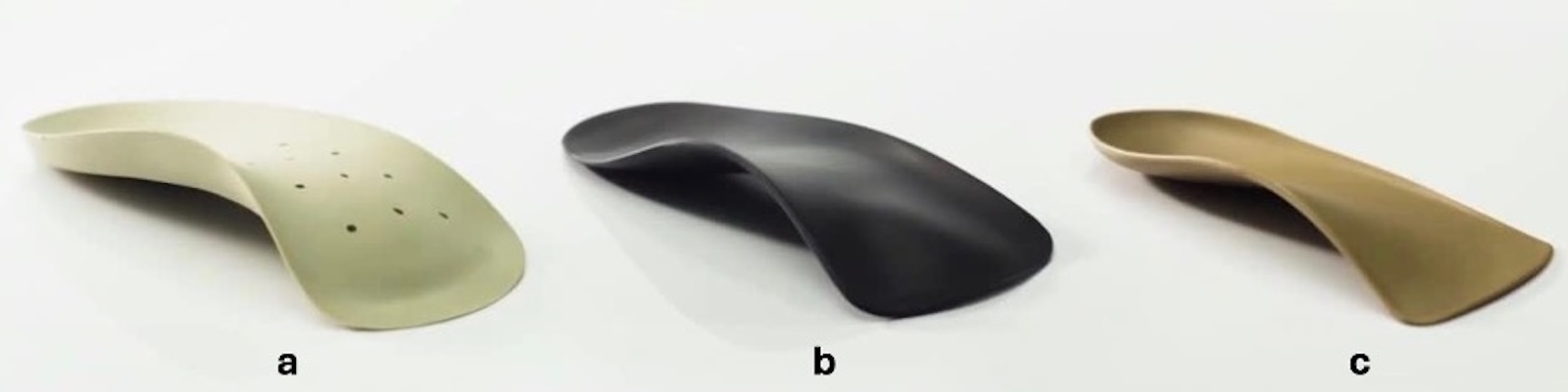

Supplement: Supplementary file 4 — Supporting File 4 [file JEO2-13-e70732-s004.jpg]

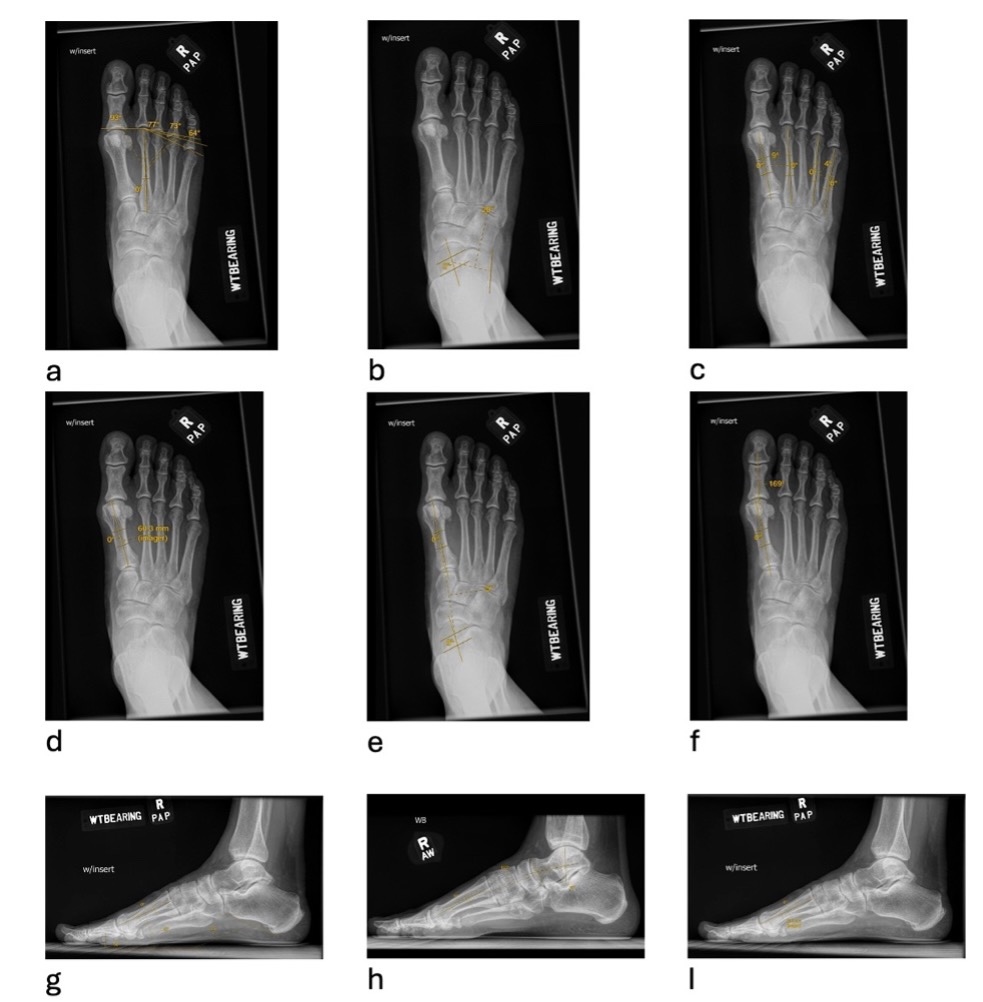

Supplement: Supplementary file 5 — Supporting File 5 [file JEO2-13-e70732-s002.jpg]

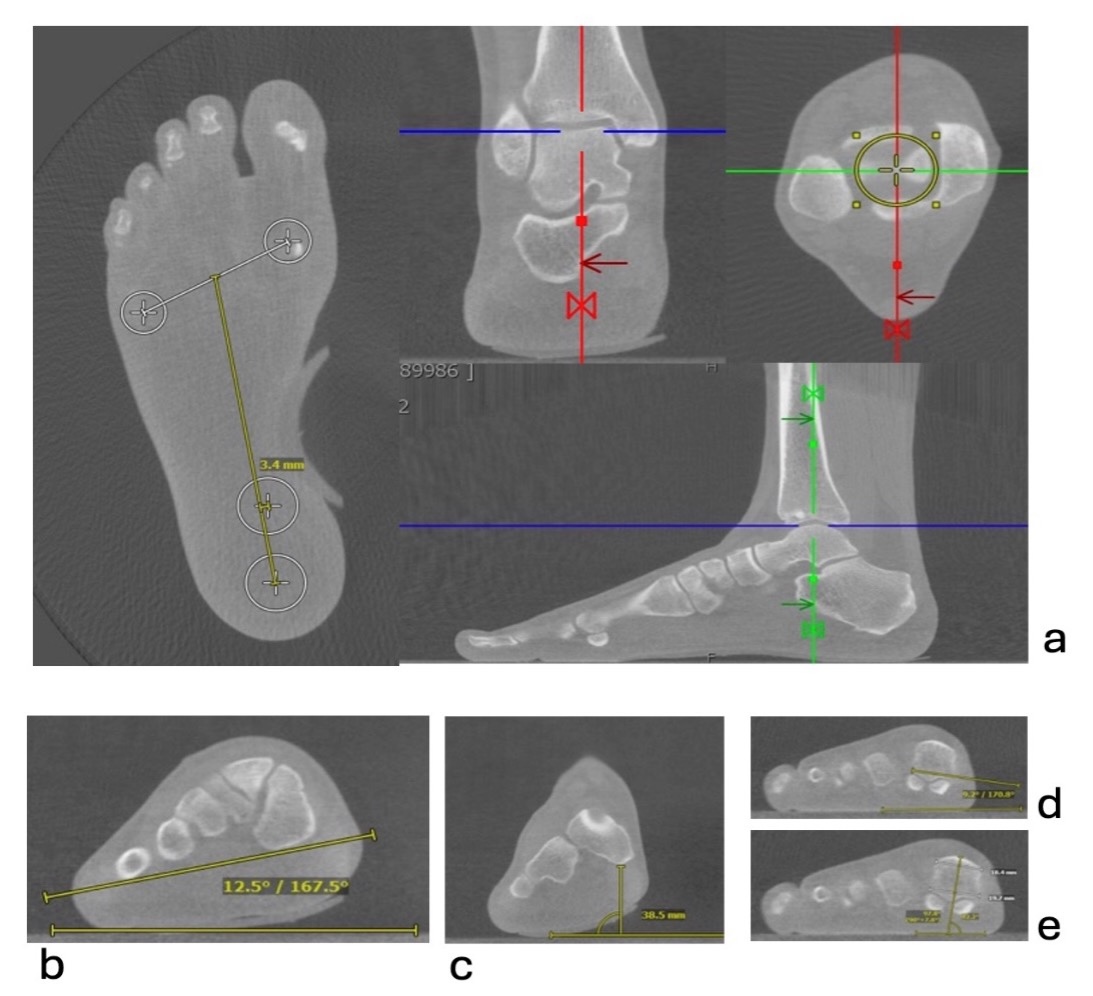

Supplement: Supplementary file 6 — Supporting File 6 [file JEO2-13-e70732-s003.jpg]
